# Genomic health data generation in the UK: a 360 view

**DOI:** 10.1038/s41431-021-00976-w

**Published:** 2021-10-19

**Authors:** Elizabeth Ormondroyd, Peter Border, Judith Hayward, Andrew Papanikitas

**Affiliations:** 1 Radcliffe Department of Medicine, University of Oxford, Oxford, UK; 2 National Institute for Health Research (NIHR) Comprehensive Biomedical Research Centre, Oxford, UK; 3 Parliamentary Office of Science and Technology, London, UK; 4 Health Education England Genomics Education Programme, London, UK; 5 Yorkshire Regional Genetics Service, Chapel Allerton Hospital, Leeds Teaching Hospitals Trust, Leeds, UK; 6 Nuffield Department of Primary Care Health Science, University of Oxford, Oxford, UK

**Keywords:** Health policy, Risk factors, Ethics

## Abstract

In the UK, genomic health data is being generated in three major contexts: the healthcare system (based on clinical indication), in large scale research programmes, and for purchasers of direct-to-consumer genetic tests. The recently delivered hybrid clinical/research programme, 100,000 Genomes Project set the scene for a new Genomic Medicine Service, through which the National Health Service aims to deliver consistent and equitable care informed by genomics, while providing data to inform academic and industry research and development. In parallel, a large scale research study, Our Future Health, has UK Government and Industry investment and aims to recruit 5 million volunteers to support research intended to improve early detection, risk stratification, and early intervention for chronic diseases. To explore how current models of genomic health data generation intersect, and to understand clinical, ethical, legal, policy and social issues arising from this intersection, we conducted a series of five multidisciplinary panel discussions attended by 28 invited stakeholders. Meetings were recorded and transcribed. We present a summary of issues identified: genomic test attributes; reasons for generating genomic health data; individuals’ motivation to seek genomic data; health service impacts; role of genetic counseling; equity; data uses and security; consent; governance and regulation. We conclude with some suggestions for policy consideration.

## Introduction

Once the preserve of specialist clinical genetics departments, patient genomic health data are now generated in mainstream medicine and from research participants. In the UK, the 100,000 Genomes Project (100kGP), a hybrid clinical/research programme, aimed to deliver genome sequencing to individual patients and families in the National Health Service (NHS) [1] while simultaneously developing a resource for research and development. 100kGP informed the recently implemented NHS Genomic Medicine Service (GMS) [2], which, through a ‘National Genomic Test Directory’ (NGTD) aims to promote consistent and equitable access to genomic tests for patients, and build a national genomic knowledge base to inform academic and industry research and development. In parallel with such initiatives, ‘Direct-to-consumer’ genetic tests (DTC-GT) can be purchased via the internet and from pharmacies. Whilst DTC-GT may have clear disclaimers about their use for healthcare decision-making, they nonetheless offer health information on which customers may base such decisions.

For individuals, genomic information can support (or reduce likelihood of) a clinical diagnosis, warn about future disease risk that might be managed through targeting of healthcare resources and behaviour change, or alert to asymptomatic disease. Generating genomic data on a large scale might prove a powerful means of improving population health through greater understanding of genomic contributions to health and disease. This potential has driven public investment in endeavours such as UK Biobank [3], AllofUs [4] in the US, and the developing Our Future Health (OFH) initiative in the UK [5]. The commercial sector has recognised the appeal of marketing genetic health data agnostic to disease presentation [6], as well as the commercial value of genomic information [7, 8]. Clearly, from a public policy perspective, there are implications for healthcare services of reporting non clinically-directed genomic health data to individuals [9].

In 2019, the UK Parliament Science and Technology Select Committee (STC) set up an inquiry into commercial genomics to collect and assess evidence to inform future policy. To contribute comprehensive evidence to the enquiry, and to explore broadly how current models of genomic health information intersect, academic researchers (EO, AP) partnered with the Parliamentary Office of Science and Technology (POST; PB), and the Health Education England Genomics Education Programme (JH). We conducted a series of five multidisciplinary panel discussions during May–July 2020. Our aim was to generate an exchange of ideas from informed stakeholders rather than a series of ‘official positions’. Representation was successfully sought from clinical, genomic science, ethical, legal, DTC-GT provider, public health and patient support groups. Discussions focused on three sources of genomic health data generation: NHS, research, and commercial sector (Table 1). A total of 28 professionals took part; many had more than one concurrent role, or had previous experience of alternative sectors. The exercise began with a strengths, weaknesses, opportunities and threats analysis in the first two meetings to generate a framework for the subsequent meetings, which the authors discussed and developed after each meeting. All meetings were conducted under Chatham House rule, [10] recorded and transcribed verbatim. A near-final draft of this manuscript was sent to all discussants; comments received were incorporated.

**Table 1:** Landscape of genomic health data generation in the UK.

Source	NHS GMS [2]	100kGP [1]	DDD [31]; NIHR BRRD [32]	UK Biobank [3]	OFH [5]	DTC [8]
Clinically directed?	Yes; according to NGTD	Yes; according to 100kGP eligibility criteria	Yes; rare disease	No	No	No
Goals	Diagnosis, treatment, research database; embedding WGS into routine care	Diagnosis, treatment, research database; NHS transformation	Diagnosis, treatment, research database	Research database	Research database for early detection of disease	Individual health and non-health related results; research database
Technology	Sequencing (whole genome or gene panel)	Sequencing (whole genome)	Sequencing (whole exome/ genome)	SNP genotyping; sequencing (whole exome/ genome)	SNP genotyping	SNP genotyping or sequencing
Individual genetic findings made available?	Yes (pertinent to presentation)	Yes (pertinent to presentation, and ‘additional findings’)	Yes (pertinent to presentation)	No	Yes (PRS)	Yes (selected)
Finance	Public	Public^a^, charitable	Public, charitable	Public, charitable	Public, commercial	Commercial
Scale	500,000 genomes	100,000 genomes	13,500 families; 13,000 participants	500,000 participants	5 million participants	Unlimited

*DDD* Deciphering Developmental Disorders.

NIHR BRRD, BioResource for Rare Diseases.

^a^Managed by Genomics England, a company set up and run by the UK Department of Health and Social Care.

This process identified a number of issues as discussed in this paper: genomic test attributes; reasons for generating genomic health data; motivation to seek genomic data; health service impacts; role of genetic counseling; equity; data uses and security; consent; governance and regulation. We conclude with some suggestions for policy consideration.

## Test attributes

In this article we use the term ‘test’ although in some contexts genomic health data generation might be considered a screen rather than a test [11].

### Technology

We considered two types of genetic test technology: sequencing of one or more genes, including the ‘whole’ genome (or the protein-coding sections, the exome), and single nucleotide polymorphism (SNP) genotyping. While sequencing ‘reads’ the genetic code and can detect individual sequence variation, SNP genotyping samples genetic variation at hundreds of thousands of specific locations across the genome. Through the NGTD [2], the GMS specifies which genomic tests are commissioned by the NHS, appropriate test technology and which patients are eligible; exome or genome sequencing are specified for some disorders. A significant proportion of rare disease develops as a result of variants in a single gene that are very rare in the population; clinical testing to investigate rare disease involves sequencing of one or a panel of genes with proven disease association. Many ‘common’ or multifactorial diseases are influenced by multiple variants that occur at higher frequency and individually have small effect size; a polygenic risk score (PRS) for a given disease can be calculated by combining information from multiple SNPs. SNP-based testing is not part of the NGTD at present. Discussants reported that most of the prominent DTC-GT companies are using SNP genotyping [12], although some offer genome sequencing, and one provider predicted that more will move towards offering sequencing. Individuals may also upload their raw data to automated ‘third party’ interpretation services for a small fee, allowing consumers to access much more data than the test provider offers [13, 14]. Research programmes use SNP arrays or genome/exome sequencing, or both; for example, UK Biobank has recently added exome sequencing data on 200,000 of its 500,000 participants [15].

In discussions, SNP genotyping of rare variants conferring high disease risk (such as *BRCA1/2* mediated cancer predisposition) was considered problematic since there may be a high proportion of false variant calling [16, 17] when analytic validation has not been performed. SNP arrays have a positive predictive value of <16% for detecting very rare variants [18]. Individuals may receive erroneous reports informing of a high risk of disease, potentially causing anxiety, wasted clinical time and expense repeating the test using appropriate technology [19]. However one participating DTC-GT provider has received regulatory authorisation for reporting on variants with high disease risk, having met a >99% accuracy and reproducibility standard for those variants.

### Clinical validity

In rare disease genetics, variant interpretation—understanding the contribution of a specific variant to disease—remains a challenge, and a focus for international efforts [20]. There is often insufficient evidence supporting variant association with disease, and interpretation can change over time as more data become available. Very large population databases are now available to aid in variant interpretation [21], with a major caveat that diverse ethnicities are under-represented. Discussants noted that increasing generation of genome sequencing data has already improved understanding of rare disease-gene relationships, and were optimistic this would continue.

Genetic disease risk prediction is often uncertain, particularly for healthy individuals receiving a rare variant result, since understanding of rare variant penetrance is based on families showing penetrant disease [22]. In the NHS, expert scientists in accredited laboratories interpret rare variants in genes with proven association with the clinical presentation, and report only those with a probability of clinical significance. In contrast, when individuals present to the NHS with ‘results’ from DTC-GT, the provenance and interpretation of data may be unclear. Further, SNP arrays allow assessment of a limited number of variants: a provider may design SNP arrays to include certain rare pathogenic variants, such as three *BRCA* variants that are over-represented in people of Ashkenazi Jewish descent [23]. However absence of a variant does not preclude the presence of another pathogenic variant not assayed by the specific SNP-genotyping, and customers with a clinical or family history of disease might be falsely reassured by a ‘negative’ result. Discussants saw this as a direct harm, compounded by misunderstandings about genetic contributions to disease, although the regulatory authorisation process completed by one DTC-GT provider had explicitly sought to mitigate false reassurance and misunderstanding through user comprehension of key concepts.

### Clinical utility

Clinical utility [24, 25] was described as ‘multi-layered’, and lacking a consistent definition. Some suggested it should encompass some element of ‘personal utility’ [26], however some argue that personal utility is contingent upon clinical validity [27]. Risk management strategies can be harmful to individuals as well as economically costly; evidence is required to inform decisions about frequency, timing and effectiveness of genomics-based interventions. Most discussants thought PRS are not ready for clinical application; further evidence is required around data interpretation, risk magnitude and risk management, communication and decision support to enact lifestyle changes [28]. While behaviour change is central to the premise of preventive risk prediction, and therefore to the utility of PRS in common disease, discussants agreed that it is difficult to measure. Several noted the lack of evidence that PRS change health behaviours [29, 30], and questioned the added value of PRS over and above well-established lifestyle advice. The view was expressed that behaviour change can be a long process; one person speculated that learning risk information at a younger age might be more effective, since people would have longer to ‘build good habits’. A further view was that patient benefit would accrue from clinicians’ responses to PRS. DTC-GT providers reported that customer surveys show that there is significant demand for genomic health information, but accepted that demand alone does not imply utility.

## Reasons for generating genomic health data

Clinically-directed genetic testing has been available in the NHS for several decades for molecular genetic investigation of a clinical presentation, and predictive testing for family members. Patient management is often informed by genetic test results. In discussions, genome sequencing was considered a powerful additional tool for rare disease genetic diagnosis in a clinical setting, including through 100kGP and other UK clinical research programmes such as Deciphering Developmental Disorders (DDD) [31] and the NIHR BioResource for Rare Disease [32]. Clinical contributors recognised the potential for genomic data to identify new gene/disease associations and refine phenotypic spectrum, and were enthusiastic about a service delivery model which combines capture of genome sequencing with phenotype data allowing aggregate and reiterative analysis such as that proposed by the GMS [2].

The dual clinical and research aims were seen as essential to the success of 100kGP, but they also created tensions, for example that patients are also research participants (consent to data collection and research access was a condition of participation), and clinicians may also be researchers, or acting on researchers’ behalf.

Discussants accepted that a key aim of commercial DTC–GT is to generate revenue, achieved in part by creating a large dataset to which access is sold for secondary research [33]. Some commercial providers mentioned specific data access partnerships with global reach, commenting that partnerships with pharmaceutical companies can be lucrative.

The explicit purpose of established population-scale research projects is to improve understanding of human health and disease; significant advancements have been made through initiatives such as UK Biobank [3], which links to participant NHS datasets from the vast majority registered with the NHS. UK Biobank provides access for proposed research that is in the public interest; researchers must undertake to publish results and to make derived data and methods available to other approved researchers [34]. OFH (previously named Early Disease Detection) aspires to create health and wealth, providing a platform for discovery research to improve the early detection or diagnosis, prevention and interventions for chronic diseases [5]. OFH will create a large dataset comprising phenotype and lifestyle data, and some genomic information derived from SNP based array technology. As envisioned by the UK Industrial Strategy Challenge Fund, OFH will partner with the NHS, potentially both for phenotype data collection and participant recruitment. OFH plans to offer some feedback of genetic information to participants. Some discussants were sceptical about the individual benefits of data collection initiatives which do not aim to answer a clinical question, and considered that maintaining realistic participant expectations should be prioritised. Some did not view large research programmes with a commercial funding component as being significantly different from DTC-GT.

## Individuals’ motivation to seek genomic information

100kGP participants took part both to derive personal or family health information, often in context of a diagnostic odyssey, and for altruistic reasons [35]. Patient representatives had a view that patients were ‘donating’ in order to further aims with intrinsic value, comparable with making a charitable donation, and on that basis had supported 100kGP. Several discussants listed the benefits for families: offering a diagnosis and promoting establishment of patient groups for mutual support, as well as hope for treatments; conversely, some discussed clinical experience of distress to patients when a diagnostic label was removed. Discussants believed there is a strong element of social solidarity in NHS-delivered services, however 100kGP was perceived as having become politicised, with one result being that the benefits for patients—likelihood of deriving clinically useful information—were overemphasised to patients and clinicians [36, 37]. Some suggested that patients in a clinical care pathway, offered appropriate genetic testing, might be less likely to seek non-NHS testing.

Primary care discussants (UK-based) reported that relatively few of their patients have so far presented with DTC-GT information; those who do tend to be relatively young, educated and affluent. One person suggested that some consumers perceive they are helping the NHS. Marketing materials presenting genetic information, particularly risk information, were considered a strength of commercial provision. However, some suggested that advertising might create demand for information that people hadn’t known they wanted. Marketing may promote tests as recreational, suitable as gifts [38], and consumers may not appreciate the potential for adverse health information. Evidence was discussed showing that DTC-GT companies expose people to more positive messages about the health benefits of test purchase and little about the limitations, accuracy and risks, which can often only be found in the contract as disclaimers [39]. Indeed, many commercial providers state that information provided does not constitute medical advice, but some discussants perceived a disconnect between sales promotion messaging and contract content.

From a healthcare perspective, a view was expressed that the NHS remains paternalistic and inadequately educates for agency in healthcare. Some suggested that patients have limited control over the information they can access in the NHS, whereas in the commercial sector consumers can theoretically access information on demand.

## Health service impacts

When genomic health data is generated for reasons other than to investigate a clinical question, a key issue for considering potential impacts on healthcare services is the type and extent of individual data made available to contributors. For example, rare variant information would warrant specialist genetics referral, genetic counselling, laboratory confirmation, clinical follow up, and family cascading.

Primary care contributors considered that health risk information can prompt useful conversations with patients. However they were wary of information that may be unclear, have uncertain evidence base, or that requires specialist referral yet is unprompted by clinical need. Primary care and clinical genetics discussants agreed that the small numbers of patients coming to the NHS with DTC-GT information take significant amounts of resources and have high expectations of a clinical response. Clinical genetics professionals reported that such patients are often anxious and confused about their disease risks. Referrals are usually accompanied by a well-presented multiple-page readout which health professionals struggle to ‘unravel, explain and undo any emotional damage’. Investigation may be hampered by lack of transparency about how the data were generated and interpreted, yet patients might have begun psychological and clinical preparation for risk-reducing surgery. Conversely, it has been argued that current testing guidelines are too conservative [23, 40, 41].

Research programmes have been encouraged to formulate a plan for returning individual results; UK Biobank for example does not provide individual feedback about information derived from analyses of data or samples [42], but may report ‘incidental’ findings detected during data collection [3]. One discussed the analogous example of reporting potentially serious incidental findings from collection of research imaging data to participants’ GPs; concerns centred on the meaning of findings, and capacity of the NHS to manage them [43].

Several discussants’ clinical experience suggested that many people struggle to understand risk, and often consider genetic information ‘deterministic’. There was a perception that patients, the public and many healthcare professionals overestimate genetic contributions to disease and the potential of genomics to explain and predict disease [44]; an example was discussed of a UK minister who publicly misinterpreted personal risk from a PRS [45]. Such beliefs could be amplified if people believe the private sector can outperform the NHS, or that personal expenditure for a test correlates with quality [46]. These factors may lead to unrealistic expectations of clinical action based on results.

Discussants talked about the importance of integrating genomics education into curricula [47]; continuing education of all health professionals was considered a crucial task requiring multidisciplinary models. Work on genomic medicine education is ongoing [48], but some felt that competence is currently patchy, potentially compromising equity of access and service delivery. Educational content should include test attributes such as analytic validity, but also ethical and social issues around genetic testing; some believed that these issues are embedded in the extensive training undergone by health professionals which provides an aspirational framework. Educating the public was also seen as important, to promote the concept that genetics is part of a longer term understanding of human health, and promote agency. Some felt this would ideally form part of the school national curriculum. One DTC-GT provider suggested that ancestry testing is a useful entry point for understanding genetics.

## Role of genetic counselling

Discussants had divergent ideas about the role of genetic counselling and whether, how, and when it should be provided. In a clinical setting, genetic counselling provides individually contextualised information about risk, aids adaptation to increasingly complex results and directs appropriate follow up care. Individual genetic test results can be important for relatives and the onus is on the individual to inform their relatives; discussing familial risk provides a forum for identifying ethical complexities and supporting communication. The role and purpose of genetic counselling in DTC-GT, or in research when patients are offered test results in return for data contribution, is unclear. Although some have encouraged genetic counseling provision in DTC-GT [49], genetic counselling arguably sits uneasily with a commercial transaction. DTC-GT providers recognised that genetic risk information can have a range of impacts; one provider explained that some companies employ genetic counsellors, but only about 3–4% of customers use this service. Another DTC-GT provider had designed education modules to be viewed before opening results; this was thought potentially useful, but dependent on the reader’s ability to understand and process probabilities.

## Equity

Good quality DTC-GT was acknowledged to have an important role where clinical services are inaccessible; one contributor suggested that recent scrutiny has improved larger DTC-GT companies’ products. Some discussants were concerned that genomic health data might increase health disparities; they felt strongly that skewing resources towards people who already access healthcare efficiently is unhelpful. While concerns were expressed that the pay-for-service model inevitably impacts equity, it was acknowledged that while equity of access is a linchpin of the NHS, not all sections of society receive equitable care. Discussants mentioned that a key aim of the GMS is to increase equity of access, but that delivery would also need equitable awareness among health professionals.

In the context of research recruiting from the population, some discussed challenges with representativeness, an acknowledged limitation of UK Biobank [50]. Many genetic databases lack ethnic diversity, which hampers efforts to generate equitable healthcare benefits. Some spoke about dilemmas experienced by indigenous peoples [51], which have led to a strong data sovereignty movement, limiting participation in genetic research [52]. Addressing ethnic representation was considered desirable for all sectors, and several people discussed ideas to increase uptake.

## Aggregate data uses

Aggregate genomic data linked to phenotypic and health outcomes data were considered to have various values. Economic value of a dataset is dependent on a number of factors including sample size and representativeness, length of follow up, type of genomic data (SNP, exome/genome sequence), depth of phenotype data, ethically approved accessability, and recontactability. Genetic databases represent a very significant commercial asset. Linkage with primary care and hospital episode data (the generation, capture and curation of which incurs significant cost to healthcare systems) clearly increases economic value. Some saw parallels between DTC-GT and ‘tech giants’ who provide a service but also generate wealth from collection and sale of data. According to DTC-GT provider discussants, potential users of DTC-GT datasets include the military, pharmaceutical and insurance companies as well as academic researchers, however a DTC-GT provider stated that their company does not share information with third parties for research purposes without the explicit consent of the customer.

Hope was expressed that large datasets could leverage improved healthcare, with benefits for individuals and society; some suggested that data-sharing in healthcare would become the norm, benefitting from partnerships with technology and possibly pharmaceutical companies. Examples discussed included using artificial intelligence to create algorithms for predictive diagnostics, achieving rare disease diagnoses, studying genetic susceptibility to infectious disease such as COVID-19 or HIV.

Several suggested that patients and the public participate in health data-sharing initiatives in order to further understanding of disease: they assume their data is contributing to the ‘greater good’ [53]. Public views around data access and uses are often strong, but nuanced [54] and difficult to articulate and capture. Commercialising data in the public sector was a concern; ‘red lines’—distinguishing the acceptable from the unacceptable—appeared when people considered how data might be used against, rather than for, public benefit: using data to discriminate or inform the benefits system, or as the basis for surveillance or insurance [55]. Some expressed the concern that many people do not feel informed enough to ask who might access their data, for what purposes and who might derive commercial benefit. One discussant stated that insurance companies are investing in DTC-GT data; this was corroborated by a DTC-GT provider who suggested that insurance companies use the concept of genetic information as a way of encouraging clients to think about their health proactively. The same person felt this underlined the importance of understanding the limitations of genetic information.

The right of ‘individuals to enjoy effective control over their personal information’ is enshrined in European Law [56], and underpins the EU General Data Protection Regulation (GDPR), the legal framework for processing (including storage) of the personal data of living individuals. GDPR includes genetic data alongside health data or biometric data as categories of data that deserve higher protection. However, since genomic data are shared—it is possible to infer identities of individuals from their relatives in genetic databases [57]—it can be challenging to determine the extent to which genomic data is ‘personal’ data, and fall within the scope of GDPR [58].

## Security

The potential for a major security breach was seen as a threat to the public’s faith in genetic testing and data storage in all sectors; the risk of a breach was considered higher if there is pressure to streamline costs at the possible expense of data protection. Cyber security is an acknowledged challenge, requiring resources to stay abreast of hackers. One DTC-GT provider discussed standards for data protection, and considered the sector has a responsibility both to meet minimum standards and to provide potential consumers with information about compliance.

## Consent

Consent is a legal basis for processing data, and all discussants recognised its central role in any endeavour collecting genomic data for future access. Many considered that informed consent in genetic testing should also include understanding the extent and implications of possible findings for the recipient and their relatives. Precisely what information should be conveyed, and how to ensure that consent is ‘informed’ were unresolved. Designing material which is comprehensive and protects an organisation yet is readable is a challenge. Discussion covered the process of gaining consent in a clinical setting, when legal contributors noted that there is now an expectation that consent conversations are individually tailored and take account of personal relevance and level of risk.

Representatives of all sectors acknowledged extensive efforts to optimise consent processes. In DTC-GT, several different types of consent approaches are in use, covering privacy, what data are collected and how they are used, and what choices are available to customers. Disclaimers in consent documents might include reservation of the right to change stated policy at any time. DTC-GT providers discussed separate research consent forms, and how they may re-contact customers to take part in specific research projects; they aim to balance ‘making consent as frictionless as possible while putting enough of a barrier there that people stop and look’. A DTC-GT provider noted that best practice is to have a consent document separate from the terms of service, and for the consent process to be overseen by an independent review body, to ensure compliance with ethical and legal guidelines. For OFH, consent materials have been co-designed with the public, following extensive focus group and interview research, in concert with developing data access and governance arrangements.

In clinical genomics research, participants are patients; consent to past, present and future health data access could be a condition of participation (such as for 100kGP) or optional as in the GMS, where the ‘research offer’ is communicated by patient choice documents. 100kGP was set up with an ethics advisory committee [1]; embedding recruitment into routine care received much deliberation. Participant information and consent forms were long and complex, and several shared the opinion that 100kGP was positioned primarily as a clinical test to patients who underappreciated its commercial nature. While consent paperwork was standardised, its complexity meant that the extent to which consent could be considered informed was dependent on the individual health professional [59] delivering an in-person interaction.

Consent for genetic testing/research participation usually occurs at a single point in time; research was discussed showing that retrospectively, patients thought they had underestimated the complexities [60]. It was felt that for consent to be informed, people need to understand implications beyond personal benefits, including the broader values of personal and aggregate data. Concern was expressed about how gaining informed consent fits alongside clinical activities, and some perceived a possible conflict between seeking informed consent and representing patients’ interests in terms of the balance of potential benefits and harms.

Consent may become problematic as soon as it loses specificity for the immediate question: for example, consent questions asking about feedback of certain types of information beyond the immediate clinical question (‘secondary’ findings), where research has shown that some participants either did not recall consent choices or recalled them incorrectly [54, 61]. Attitudes towards the types of health information people might want to learn might change over the life course. Consent forms that combine questions asking for affirmation, with questions asking participants to make a choice, might have the unintended consequence that more time and effort is spent thinking about the latter rather than the larger context of the project.

The impression that generating commercial profit from research data (with or without public benefit) is unpalatable to the public led to discussions about transparency and trust. Some considered that the commercial component of 100kGP presented an ethical dilemma; it was suggested that any commercial company—including when set up to incorporate ethical principles—could be sold on. A view was also expressed that if the state has invested in creating a dataset (recruitment, genotyping and phenotyping, data input and curation), prioritising benefits to the NHS is reasonable.

The ‘broad consent’ model [60, 62, 63] also relies on participant consent at a single time, but many factors can change the use, and usefulness of the data: data interpretation, technology, governance laws, acceptable uses and commercial partners were listed as examples. Equally, social and environmental circumstances may change abruptly, as in the onset of a pandemic, or there may be slower changes to the ‘social contract’. None of these factors can be known or predicted at the time of consent, and some saw broad consent as a kind of ‘get-out clause’. Seeking re-consent was seen as respectful of autonomy but very resource intensive.

The challenges discussed above suggest that consent cannot be the sole means of ensuring that data uses are ethically valid, and that data contributors’ values are respected. Consent is necessary, but not sufficient; it forms one component in the framework in which the test sits. The concept of trust in those frameworks is critical [64]; several discussants with clinical experience considered that 100kGP benefitted from the trust accrued by the NHS as a beneficent service provider. Participants were ascertained and recruited by NHS personnel in teams often known to them [54]; recruitment materials carried ‘those three letters in the little blue box’, and ‘assumed [their donation] is going to the greater good’. Publicity materials were perceived to have emphasised the personal benefits of participation, including families who had been provided with a valued diagnosis. Some felt that this message had been disingenuous. For OFH, still in planning stages, reaching an agreement to partner with the NHS for recruitment and data gathering, was recognised as critical.

## Governance and regulation

Trustworthiness was considered to encompass ideas of public (including future public) benefit, evidence-based standards, governance [65], transparency, consistency and communication. Trustworthiness applies to people, infrastructure and processes. Some felt that the sustainability of genomic research depends on public trust, and that data access needs appropriate governance. In the research environment, participants may be told that data uses are unpredictable, but that there are usage restrictions, and discussants mentioned that some very sensitive datasets—for example where ethnic tensions are involved—are not released. Regulating access can happen at several levels, including data access agreements, by affiliation, project review, restriction to secure servers, as well as having data access committees who make strategic decisions. As an example of an adverse event, discussants talked about the sharing with Google DeepMind of identifiable medical information of millions of UK patients through a data-sharing agreement with an NHS Trust without appropriate consent [66].

One commercial sector provider acknowledged the potential that the prospect of ‘great revenues’, might tempt some to take shortcuts around handling of data and ethical frameworks, and would welcome government-mandated mechanisms to protect consumers. Another commercial provider stated a preference for a balanced approach to regulation, recognising that forthcoming European Union In Vitro Diagnostic Medical Device regulations (IVDR) [67] are challenging for DTC-GT providers. The need for regulation was re-iterated by submissions to the STC committee hearing. Discussants suggested that the scope of regulation should include data flows, data access, test quality, analytic and evidence-based clinical validity, and variant interpretation. In view of GDPR giving individuals rights over their data, regulation of information and the uses consumers can make of ‘their’ information was considered less feasible. IVDR requires manufacturers to provide evidence for analytical and clinical performance, while leaving member states free to determine how informed consent and genetic counselling should be provided [68]. From January 2021, all medical devices including in vitro diagnostic medical devices placed on the Great Britain market need to be registered with the Medicines and Healthcare products Regulatory Agency (MHRA) [69]. The recent House of Commons Science and Technology Committee report makes several recommendations for regulation, and urges the Government to set out a specific timeframe in which it intends to review the case for introducing new regulations for genomic tests provided directly to consumers [70].

## Conclusions

Generation of genomic health data in the UK involves public and private sector interaction at multiple points, and care is required to balance rights and protections of patients, publics and public healthcare systems. Despite the concerns outlined in this article, there was broad consensus that plurality in provision of genomic health information is inevitable. A publicly funded health system such as the NHS requires buffering against tests which provide information of lower quality or benefit. Protecting consumers requires that tests marketed commercially should, at a minimum, measure what they claim to measure, make accurate claims, and be safe [9].

DTC-GT and population research tests are not part of routine healthcare: their role, and that of PRS in healthcare is uncertain. More research is needed around the clinical utility of different types of risk information including PRS. There is a danger that tests that are not clinically indicated could overdiagnose, transforming ‘well’ people, whose risk is low, into patients receiving NHS care. In order to provide equitable care the NHS prioritises clinical need; recent guidance [71] represents an attempt to manage DTC-GT in the NHS. We also highlight the need for protection of individual healthcare data, and for its use to be transparent and to respect public preferences. There was a consensus that governance and ongoing review are required to ensure trustworthiness and maintain the trust of current and future data contributors. We make the following suggestions for policy attention:

1. International regulatory standards for genetic testing, including test technology, variant calling and reporting, should apply to all individual genomic data that are reported.
2. Patients, research participants and consumers should expect clear information about the test and results. Information should be evidence-based, regularly reviewed and updated, and purported benefits and limitations responsibly balanced. Information about data uses, privacy and security should also be provided, with options for full withdrawal of data, including de-identified data.
3. Data collection initiatives benefiting from public sector investment, or individual health data harvesting, should prioritise and resource efforts to understand and respect public opinion, put in place transparent and robust governance structures, and include a principle of re-investment of revenue into public healthcare and health promotion.
4. Consideration should be given to models of joint provision, for example a ‘third way’, in which commercial providers fund an independent organisation staffed by trained and professionally regulated personnel such as clinical scientists and genetic counsellors. Under this model, individuals could be triaged and those who meet clinical risk criteria managed within the NHS. Clinical outcomes data on rare variants identified outside the context of clinically ascertained families is required to inform clinical utility, and consent should be sought for data capture.

## Data Availability

Data sharing not applicable to this article as no datasets were generated or analysed during the current study.
